# The COMIX polarimeter: a compact device for XUV polarization analysis

**DOI:** 10.1107/S1600577522004027

**Published:** 2022-05-19

**Authors:** Matteo Pancaldi, Christian Strüber, Bertram Friedrich, Emanuele Pedersoli, Dario De Angelis, Ivaylo P. Nikolov, Michele Manfredda, Laura Foglia, Sergiy Yulin, Carlo Spezzani, Maurizio Sacchi, Stefan Eisebitt, Clemens von Korff Schmising, Flavio Capotondi

**Affiliations:** a Elettra-Sincrotrone Trieste SCpA, 34149 Basovizza, Italy; b Department of Physics, Freie Universität Berlin, 14195 Berlin, Germany; c Max Born Institute for Nonlinear Optics and Short Pulse Spectroscopy, 12489 Berlin, Germany; d Fraunhofer Institute for Applied Optics and Precision Engineering IOF, Albert-Einstein-Straße 7, 07745 Jena, Germany; e Sorbonne Université, CNRS, Institut des NanoSciences de Paris, INSP, 75005 Paris, France; f Synchrotron SOLEIL, L’Orme des Merisiers, Saint-Aubin, BP 48, 91192 Gif-sur-Yvette, France; g Institut für Optik und Atomare Physik, Technische Universität Berlin, Strasse des 17 Juni 135, 10623 Berlin, Germany

**Keywords:** extreme-ultraviolet polarimetry, free-electron laser, pump–probe experiments, magnetic materials

## Abstract

We report on the characterization of a novel extreme-ultraviolet polarimeter based on conical mirrors to simultaneously detect all the components of the electric field vector for extreme-ultraviolet radiation in the 45–90 eV energy range. The device has been characterized using a variable polarization source at the Elettra synchrotron, showing good performance in the ability to determine the radiation polarization. Furthermore, as a possible application of the device, Faraday spectroscopy and time-resolved experiments have been performed at the Fe *M*_2,3_-edge on an FeGd ferrimagnetic thin film using the FERMI free-electron laser source. The instrument is shown to be able to detect the small angular variation induced by an optical external stimulus on the polarization state of the light after interaction with magnetic thin film, making the device an appealing tool for magnetization dynamics research.

## Abstract 2

The COMIX instrument, a novel compact polarimeter for XUV light based on conical mirrors, has been tested and characterized. Through a time-resolved experiment on a ferrimagnetic thin film, it is shown that the device has the potential to become a versatile tool for research in femtomagnetism.

## 1. Introduction

With the recent progress in generation and control of arbitrary polarization states of radiation in the extreme ultraviolet (XUV) and soft X-ray spectral ranges, both at laser-based high harmonic generation sources (Fleischer *et al.*, 2014 ▸; Kfir *et al.*, 2015 ▸; Ayuso *et al.*, 2019 ▸) and at free-electron laser (FEL) facilities (Allaria *et al.*, 2014 ▸; Lutman *et al.*, 2016 ▸; von Korff Schmising *et al.*, 2017 ▸; Tschentscher *et al.*, 2017 ▸), increasing attention has been devoted to the investigation of chiral and circular dichroic properties of matter. In particular, in solid-state research of magnetic materials, access to the element-specific response via magnetic circular dichroism has been shown to be essential in order to gain a fundamental understanding of the intrinsic microscopic processes (Kirilyuk *et al.*, 2010 ▸). Prominent and important examples include all-optical switching in transition metal (TM) and rare earth (RE) alloys, such as, for example, FeGd (Stanciu, Hansteen *et al.*, 2007 ▸; Radu *et al.*, 2011 ▸), CoTb (Bergeard *et al.*, 2014 ▸), FeTb (Khorsand *et al.*, 2013 ▸), and very recently the first experimental evidence for optical-induced spin transfer between two distinct sites in a multi-component magnetic system (Hofherr *et al.*, 2020 ▸; Willems *et al.*, 2020 ▸). Another application of polarimeters in the soft X-ray range is the investigation of natural birefringence in chemistry (Palmer *et al.*, 2011 ▸) or solid-state physics (Mertins *et al.*, 2004 ▸). The latter was demonstrated for graphite yielding extraordinarily large polarization rotations of up to 90° at the carbon *K*-edge. As a consequence, several XUV/soft X-ray polarimeters have been developed based on Rabinovitch reflection geometry (Rabinovitch *et al.*, 1965 ▸; Schäfers *et al.*, 1999 ▸; Yamamoto & Matsuda, 2017 ▸), on electron time-of-flight (e-TOF) detection of the angular distribution of emitted photoelectrons by rare-gas atoms (Viefhaus *et al.*, 2013 ▸; von Korff Schmising *et al.*, 2017 ▸), and, more recently, on beam splitting and polarization-sensitive balanced photodetection of orthogonal electric field components similar to the Wollaston scheme in visible light (Caretta *et al.*, 2021 ▸). All of these devices present several advantages and some drawbacks. For example, polarimeters based on Rabinovitch reflection geometry have a large energy range of applications, spanning from XUV to soft X-ray thanks to the single reflection scheme and the use of high-reflectivity multilayer mirrors. However, to fully characterize the beam polarization, a 360° rotation of the analyser mirror and detector photodiode is necessary, in order to map the polar dependence of the beam reflectivity. As a consequence, the measurements are quite time consuming. On the other hand, polarimeters based on the polar distribution of photoelectrons in the gas phase allow for single-shot measurements of the beam polarization state at FEL facilities (Allaria *et al.*, 2014 ▸; von Korff Schmising *et al.*, 2017 ▸; Ferrari *et al.*, 2015 ▸; Lutman *et al.*, 2016 ▸), but they require expensive instrumental hardware due to the acquisition of several signals in coincidence, and they are limited in energy range by the photoionization cross-section of the detection gas. Finally, differently from Rabinovitch-based polarimeters, a recently developed device based on mirror beam-splitting and balanced detection (Caretta *et al.*, 2021 ▸) combines the advantage of single-shot detection with fixed mechanical components, but the beam pointing stability is still a limiting factor, since it uses two microchannel plates (and not a CCD detector) to record the intensity of the two orthogonal electric field components.

In this paper, we present a novel approach using a compact polarimeter based on two conical mirrors for XUV light (COMIX) capable of detecting all the components of the electric field vector in a single measurement, so allowing for polarization analysis in the XUV region of the electromagnetic spectrum. The COMIX combines the main advantages of the above-described polarimeters, *i.e.* (i) the possibility to determine the polarization state of XUV light thanks to the polar dependence of mirror reflectivity, (ii) the possibility to work in single-shot mode for time-resolved applications at FEL sources, and (iii) the use of a relatively inexpensive experimental hardware with respect to more sophisticated schemes based on gas ionization.

The paper is organized as follows. In the first part, we describe the working principle of the COMIX polarimeter and its optical characterization performed at a synchrotron radiation source. We demonstrate the core capability of the device, *i.e.* the ability to simultaneously detect all the components of the electric field vector in a range of photon energies from 45 to 90 eV. We also report that the main limiting factor of the current device prototype is the optical quality of the mirrors used for building the polarimeter. In the second part of the paper, we present a possible application of the COMIX device using FEL radiation for studying the ultrafast magneto-optical properties of an FeGd ferrimagnetic thin film. Even if still limited by the mirror surface roughness, we show that the COMIX polarimeter is able to perform precise magneto-optical measurements in polar Faraday geometry, allowing for a spectroscopic analysis across the Fe *M*_2,3_ absorption edge (∼52.8 eV). Finally, we show that the device is able to successfully detect small variations (of the order of 0.17°) in the light polarization state induced by an external optical stimulus, making the COMIX polarimeter an appealing device to probe the polarization state of XUV radiation during light–matter interaction in time-resolved measurements.

## 2. Device characterization at the synchrotron

**Figure 1:**
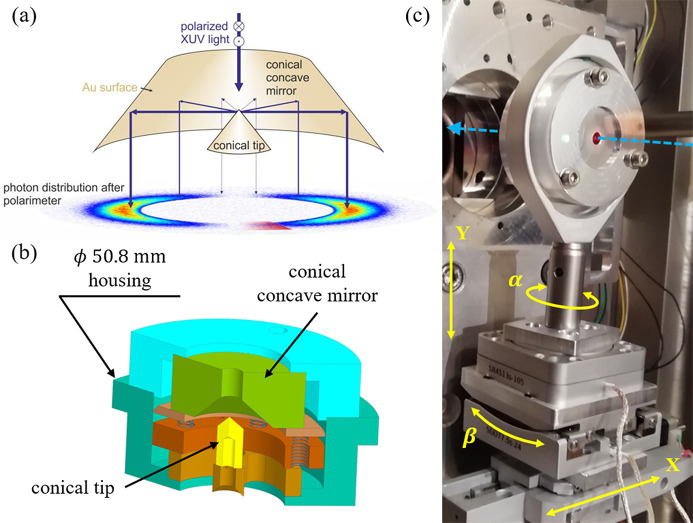
(*a*) Schematic view of the COMIX polarimeter. Due to the cylindrical symmetry, the transmitted intensity distribution depends on the polarization state of the incident beam. (*b*) Mechanical drawing of the COMIX polarimeter prototype, which can be mounted in standard optomechanical stages. (*c*) Four-axis motorized goniometer to precisely align the conical tip apex with respect to the XUV beam. The light blue dashed arrow shows the axis of the XUV beam, directed towards the CCD camera (not shown).

Despite the similarity with a Schwarzschild objective (Lan & Twa, 2019 ▸), the COMIX polarimeter is composed of two flat-type conical mirrors (provided by Thorlabs Inc.) with a common axis of cylindrical symmetry, as schematically shown in Fig. 1 ▸(*a*). After entering the device through a 2 mm-diameter aperture opened in the retro-reflecting concave element, the incoming XUV light is first reflected around 360° by the inner conical tip, then by the outer concave mirror, which forms a collimated ring-like beam propagating in the forward direction towards the two-dimensional CCD detector. The polar dependence of the collected intensity pattern from the input polarization state is determined both by the cones’ surface coating and by the cones’ aperture angle. The COMIX polarimeter prototype is designed with an aperture angle of 42°, corresponding to Brewster’s angle for a reflection off a gold-coated surface at a beam energy resonating with the Fe *M*_2,3_-edge. At this energy, the reflectance of *s*-polarized light after two reflections is 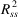 = 1.0 × 10^−2^, whereas for *p*-polarized light it is 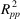 = 1.6 × 10^−4^, corresponding to a device extinction ratio of 65 between the two polarization states (Henke *et al.*, 1993 ▸). The polar modulation of the transmitted intensity ring can be estimated within the Jones matrices formalism (Fowles, 1989 ▸). As a function of the polar angle ϑ around the centre of the pattern, the transmitted intensity *I*_t_ is defined by

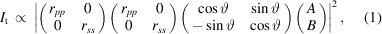

where *A* and *B* are complex numbers representing an arbitrary polarization state, and *r*_*ii*_ is the reflectivity for *i*-polarized light (*i* = *s*, *p*). Neglecting *r*_*pp*_, it turns out that

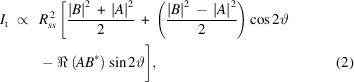

where 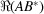 is the real part of the *AB* ^*^ product and 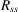 = 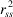 . Hence, the polar modulation of the transmitted intensity ring shows a 180° periodicity. The same principle can be applied to an energy range around the optimal design energy (as described in the following sections), making the gold-coated COMIX polarimeter an interesting device for performing magneto-optical Faraday measurements at Fe, Co and Ni *M*_2,3_-edges (Valencia *et al.*, 2006 ▸).

With respect to other XUV polarimeters (von Korff Schmising *et al.*, 2017 ▸), one of the advantages of the COMIX device is its compactness. As shown by the mechanical drawing in Fig. 1 ▸(*b*), the outermost diameter of the aluminium housing holding the conical mirrors in place is only 50.8 mm, which allows the use of standard two-inch optomechanical mounts. Moreover, due to its limited weight, it is very easy to assemble a vacuum-compatible motorized stage based on piezo-driven motors with all the degrees of freedom needed for a proper device alignment. As shown in Fig. 1 ▸(*c*), we used two stepper motor linear translation stages to control, with micrometric precision, the placement of the COMIX polarimeter in the *XY*-plane perpendicular to the beam axis, while two piezoelectric stages allow the tuning of the α and β angles. It is worth noticing that, in the present version of the polarimeter, the internal alignment of the two optical elements forming the instrument is performed mechanically during the assembly, and the motorized stages of the four-axis goniometer are used for aligning the tip apex of the inner conical mirror with respect to the input beam wavefront.

To thoroughly test the COMIX capabilities, measurements in the 45–90 eV energy range have been performed at the Circular Polarization (CiPo) beamline of the Elettra synchrotron (Derossi *et al.*, 1995 ▸), by making use of the IRMA experimental chamber (Sacchi *et al.*, 2003 ▸). Through an electromagnetic elliptical wiggler (EEW), the CiPo beamline delivers XUV light with variable polarization (linear and circular). Moreover, the horizontal and vertical magnetic fields of the EEW can be set independently, in order to finely control the variation of polarization state. The desired wavelength is selected by using a spherical-grating monochromator (SGM) and a pair of slits placed before and after it. An Andor iKon-L CCD camera (with a 13.5 µm pixel size) placed approximately 200 mm downstream of the COMIX polarimeter was used for recording the transmitted intensity pattern.

**Figure 2:**
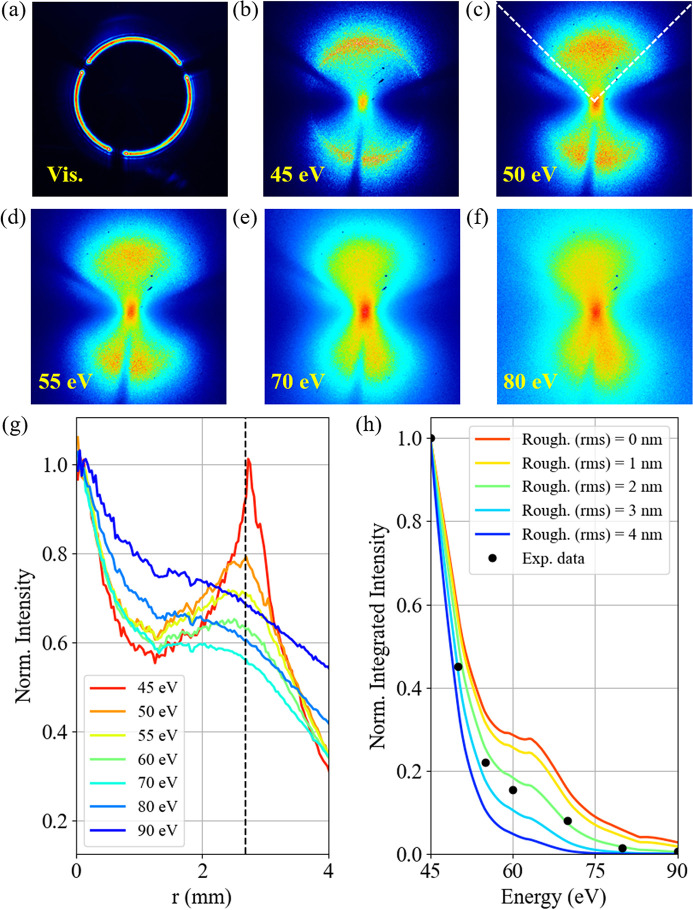
(*a*) Recorded intensity pattern obtained with unpolarized visible light. A circular ring-like structure is clearly visible. (*b*)–(*f*) Recorded intensity patterns obtained with LH polarized light of increasing photon energy, as stated in the labels. The colour bar limits are different for each panel, and they have been chosen to better highlight the features of interest. (*g*) Radial profiles extracted from the region contained between the two white dashed lines in (*c*). All the profiles are normalized to the intensity at the centre of the image (*r* = 0). The vertical black dashed line highlights the average radius of the circular structure in (*a*). For increasing energy, the amplitude of the peak corresponding to the ring-like feature decreases, while its width is enhanced. (*h*) Normalized integrated intensity as a function of the photon energy. The black dots represent the experimental data, while the continuous coloured lines show the predicted energy dependence of the COMIX transmittance for mirror roughnesses in the 0–4 nm (r.m.s.) range.

In order to align the COMIX polarimeter, we turned off the EEW emission to obtain unpolarized bending-magnet radiation, which was then transmitted by the SGM grating at zero order and filtered by an optical window. A proper alignment of the COMIX produces the nearly uniform, well defined ring shape shown in Fig. 2 ▸(*a*). The three 120°-spaced dark regions inside the bright ring are the shadows of the mounting frame for the inner conical mirror. By construction design, the radius of the ring is only determined by the distance between the conical mirrors, which is fixed. Hence, in our proof-of-concept experiment, we expected to obtain annular patterns of roughly the same radius, irrespective of the photon energy. Fig. 2 ▸(*b*) shows the pattern obtained by selecting an energy of 45 eV when the radiation is linearly polarized in the horizontal plane (LH polarization). In this measurement, higher orders from both the EEW and the SGM were removed by an Al filter inserted in the photon transport line, and a 20 µm pinhole was placed 400 mm upstream of the COMIX polarimeter to generate a secondary source providing an illumination spot size of about 400 µm (full width at half-maximum, FWHM) for the inner conical mirror at 60 eV. Since the polarization is linear horizontal, the polarimeter produces the expected intensity modulation, *i.e.* two bright lobes along the vertical direction (*s*-polarization scattering) separated by two darker regions placed at 90° with respect to the intensity maxima (*p*-polarization scattering). One can also notice that, differently from the visible-light image of Fig. 2 ▸(*a*), the ring-like shape is smeared out by diffuse intensity, mainly due to the surface roughness of the COMIX prototype, which tends to concentrate photons in the centre of the pattern. This effect becomes more evident when increasing the photon energy, as shown in Figs. 2 ▸(*c*)–2(*f*). As a general trend, for all the energies a clear two-lobed structure can be identified in the output intensity pattern, demonstrating that the polarimeter is able to detect the linear polarization state of the incoming radiation in the considered energy range. However, the ring-like feature becomes fainter when increasing the photon energy, and the intensity tends to become more concentrated in the central part of the pattern resulting in a ‘bow-tie’ shape with a bright spot in the middle, as shown in Fig. 2 ▸(*f*). Despite this, the ring-like structure has the same average radius of the ring shown in Fig. 2 ▸(*a*), as can be seen from the normalized radial profiles of Fig. 2 ▸(*g*), which are extracted from the region contained between the two white dashed lines in Fig. 2 ▸(*c*). Fig. 2 ▸(*g*) also shows that the width of the ring-like structure increases as a function of the photon energy, and the relative intensity of the diffuse background light increases as well, suggesting that, with the current optical elements, the surface quality is the limiting factor and the parameter to be improved in order to extend the capabilities of the COMIX polarimeter beyond 90 eV. These measurements also allow obtaining an estimate of the gold-coated conical mirrors roughness. Indeed, by summing the counts over all the pixels of the collected images, the total transmitted intensity by the COMIX can be evaluated as a function of the photon energy, as shown in Fig. 2 ▸(*h*). For each energy, the integrated intensity has been normalized to the incident photon flux, the image exposure time, the CCD quantum efficiency and the number of generated electrons in Si, considering an electron–hole pair creation energy of 3.65 eV (Desjardins *et al.*, 2020 ▸). The total normalized intensity values [black dots in Fig. 2 ▸(*h*)] can then be compared with the expected total intensity calculated by integrating equation (2) over the polar angle ϑ. The result, which turns out to be proportional to 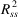 , is evaluated by taking into account the mirror roughness (Henke *et al.*, 1993 ▸), as shown by solid lines in Fig. 2 ▸(*h*). From this analysis, we estimate that the COMIX polarimeter is characterized by an average total mirror roughness of the order of 2–3 nm (r.m.s.). As reported in Fig. S1 of the supporting information, an atomic force microscopy image performed on the surface of the conical substrate before the gold coating deposition shows an average roughness of approximately 2 nm (r.m.s.). This roughness is the main reason determining the poor optical quality of the device at XUV wavelengths.

**Figure 3:**
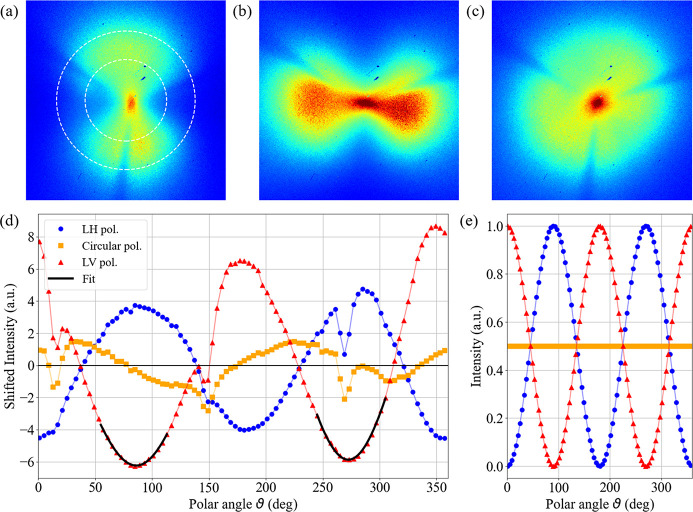
Intensity pattern recorded at 75 eV photon energy with nominally (*a*) LH, (*b*) LV and (*c*) circular polarization. The three images share the same colour bar and have been normalized to the incident beam intensity and to the exposure time. (*d*) Polar profiles obtained for images (*a*)–(*c*) within the region of interest depicted in panel (*a*). The glitch discontinuities at 30, 150 and 270° are due to the shadow of the mounting frame for the inner conical mirror. (*e*) Calculated polar profiles for ideal polarization states obtained from equation (2), to be compared with panel (*d*).

For testing the sensitivity of the COMIX polarimeter to polarization changes, we fixed the photon energy to 75 eV while varying the beam polarization. To do so, we started with nominally LH polarized light, by turning on the vertical magnetic field of the EEW. The intensity pattern is reported in Fig. 3 ▸(*a*). Then, only the horizontal magnetic field was turned on, and light linearly polarized in the vertical plane (LV polarization) was obtained, as shown in Fig. 3 ▸(*b*). Once the two linear polarizations were established and recorded, the relative amount of the two components was changed by progressively reducing the amplitude of the horizontal magnetic field and increasing accordingly the vertical magnetic field, in order to maximize the incident beam intensity. In general, an elliptically polarized beam is obtained, as shown in the video in the supporting information. Fig. 3 ▸(*c*) shows an intermediate case, which corresponds to a nominally circularly polarized light according to the beamline design parameters. To better quantify the quality of the polarization-dependent intensity patterns, we extracted polar profiles in the region of interest shown in Fig. 3 ▸(*a*), which corresponds to the region in which the ideal ring produced by the COMIX should be. Those profiles are shown in Fig. 3 ▸(*d*). To retrieve the relative shift, the curves can be fitted with an *f*(ϑ) function describing a 180°-period modulation (von Korff Schmising *et al.*, 2020 ▸), as also obtained from equation (2),

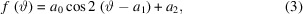

where the parameter *a*_1_ is the phase shift we would like to assess (corresponding to the rotation of the intensity pattern). Actually, for each curve only the two minima were independently fitted in a range of ±30° around each minimum, as shown by black solid lines in Fig. 3 ▸(*d*) for LV polarization. Then, the final shift value was defined by averaging the results obtained for the two minima. According to this procedure, the relative rotation between the intensity patterns in Figs. 3 ▸(*a*) and 3(*b*) results to be (92.2 ± 0.3)°, *i.e.* very close to the 90° value expected for two perpendicular linear polarizations. The slight difference is associated with the beamline performance, when using the nominal parameters of the EEW source for setting LH and LV polarization emission.

Furthermore, the orange curve in Fig. 3 ▸(*d*) has been extracted from Fig. 3 ▸(*c*), corresponding to nominally circular polarization for the beamline. To better understand the general behaviour, let us focus on an ideal case. As shown in Fig. 3 ▸(*e*), the intensity pattern produced by linear polarization should show a regular 180° periodicity, with a 90° phase shift between perpendicular polarizations. The blue and red curves in Fig. 3 ▸(*e*) have been obtained from equation (2) by considering *A* = 1, *B* = 0 (LH polarization) and *A* = 0, *B* = 1 (LV polarization), respectively. In the case of circular polarization, the intensity is equally distributed around the pattern centre, as can be verified by evaluating equation (2) with *A* = 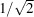 and *B* = 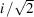 . However, the plots in Fig. 3 ▸(*d*) show two main deviations from the predicted theoretical trends. First of all, in the case of LV polarization, the maximum at around 170° has a slightly lower amplitude than the maximum at around 350°. This small discrepancy can be ascribed to a COMIX misalignment with respect to the input beam intensity maximum. Indeed, the positioning of the tip apex of the inner conical mirror is critical in determining how the incoming beam intensity is split between the two bright lobes. Experimentally we observe that, for focused beams of a few hundreds of micrometres in FWHM, variation as small as 10 µm in positioning the COMIX tip apex are able to slightly unbalance the intensity between the bright lobes. This issue currently prevents a full quantitative analysis of the polarization state, since the polarization ellipticity cannot be readily evaluated (for further details about the analysis of the polarization ellipticity see Fig. S2 of the supporting information). It is worth noticing that this position-sensitive effect can be mitigated, at the expense of the total incoming fluence, by reducing the size of the input pinhole (currently 20 µm) used as secondary source, in order to generate a wider Airy diffraction pattern for illuminating the inner cone of the COMIX polarimeter. As a reference, for the experimental geometry described above, a 4 µm pinhole generates an Airy diffraction disc of 2 mm in diameter (FWHM) for a photon energy of 60 eV at a distance of 400 mm, matching the input aperture of the COMIX. Secondly, the circularly polarized case still possesses a polar modulation around the centre, which is shifted by approximately 45° and with reduced amplitude (approximately 20%) with respect to the linearly polarized cases. This modulation is not related to the COMIX performance, but it is due to a slight misalignment between the undulator and beamline axes, combined with a contamination from the bending magnet emission (Frassetto *et al.*, 2019 ▸). Even if the problem has been identified, we could not correct it during the time of the experiment.

However, already in the present form, the COMIX polarimeter allows for precisely determining the degree of polarization rotation, as confirmed in the next section.

## 3. Free-electron laser characterization using a magnetic sample

**Figure 4:**
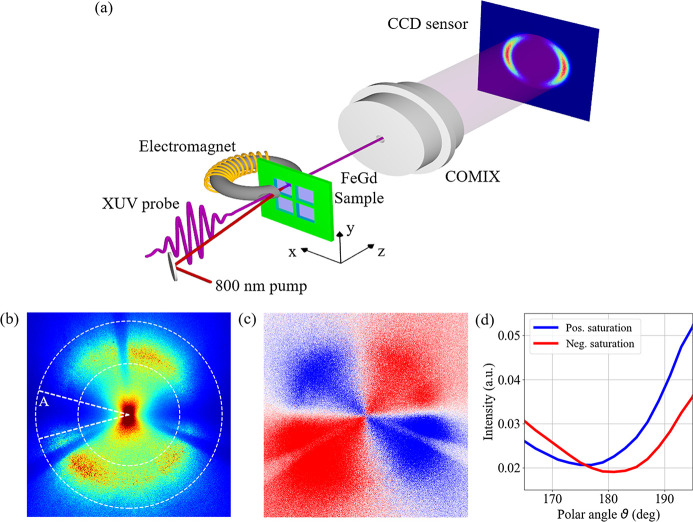
(*a*) Sketch of the experimental setup for time-resolved pump–probe measurements. (*b*) Intensity pattern recorded with LH polarized light tuned to 52.8 eV when the FeGd thin film was saturated along the *z* axis (positive saturation). (*c*) Difference signal between panel (*b*) and the corresponding image obtained saturating the sample in the opposite direction (negative saturation). By doing so, the common background can be removed, and the relative changes are highlighted. (*d*) Polar profiles obtained in a ±15° range around the intensity minimum labelled by A for panel (*b*) and for the corresponding image obtained at negative saturation. After fitting, a relative Faraday rotation of (4.6 ± 0.4)° has been evaluated.

The characterization performed at the CiPo beamline allowed us to test the core capabilities of the COMIX polarimeter itself, showing its functionality and working energy range. In this section, we concentrate on a possible application of the device for studying the static and dynamical properties of magnetic thin films using FEL radiation. In this particular application, the COMIX polarimeter has been exploited to detect the Faraday rotation of a linearly polarized XUV beam induced by a ferrimagnetic thin film. The experiment was carried out at the DiProI endstation (Capotondi *et al.*, 2013 ▸), one of the instruments available at the FERMI FEL user facility (Allaria *et al.*, 2012 ▸). A sketch of the experimental setup is shown in Fig. 4 ▸(*a*). Linearly polarized XUV light in the 50–60 eV energy range is transmitted at normal incidence through a ferrimagnetic thin film deposited on top of a 50 nm-thick Si_3_N_4_ membrane, and the direct beam is intercepted by the COMIX polarimeter. The resulting intensity pattern is finally recorded by an in-vacuum Princeton MTE2048 CCD camera placed approximately 200 mm after the COMIX. The sample consists of a Ta(3)/Fe_76_Gd_24_(40)/Pt(3) stack (thickness in nm) featuring perpendicular magnetic anisotropy. This material has been chosen since it has a relatively large Verdet constant in the XUV range (Yamamoto *et al.*, 2015 ▸) and, being a common material in the all-optical switching field (Stanciu, Hansteen *et al.*, 2007 ▸; Radu *et al.*, 2011 ▸), it is very well characterized in the literature. To control the magnetic state of the sample, an electromagnet was integrated into the setup, providing a variable external magnetic field up to 250 mT perpendicular to the sample surface.

In order to perform time-resolved experiments, the FEL probing pulses (with duration of 70 fs and repetition rate of 50 Hz) were focused down to a spot size of approximately 250 µm × 250 µm by the active optics of the endstation (Raimondi *et al.*, 2013 ▸, 2019 ▸). To excite the sample, a near-infrared 800 nm laser beam (with 60 fs pulse duration) synchronized with the FEL radiation (Danailov *et al.*, 2014 ▸) is focused down to a spot size of approximately 450 µm × 450 µm and directed on top of the probed sample region. In order to avoid saturation of the detector by the pumping near-infrared laser radiation, the CCD sensor was protected by a 200 nm-thick Al filter.

Fig. 4 ▸(*b*) shows the intensity pattern obtained as an average of ten images (400 FEL shots per image) after the sample interaction with LH polarized light tuned to 52.8 eV, corresponding to the Fe *M*_2,3_-edge (Valencia *et al.*, 2006 ▸). The sample was kept saturated with a fixed magnetic field, in order to maximize the polarization rotation. Even if, due to the sample transmittance of about 3%, we collected data in multi-exposure mode, we would like to stress that, as shown in Fig. S3 of the supporting information, the COMIX polarimeter is able to collect polarization-dependent intensity patterns in single-shot fashion, which can be helpful for precisely optimizing the FEL emission and characterizing its polarization stability. Comparing a sequence of 20 successive single FEL shots, we estimate a shot-to-shot polarization instability of 0.8° (FWHM) with respect to the nominal linear horizontal polarization direction, as described in Fig. S3(*e*). As obtained for the synchrotron measurements in Fig. 2 ▸, a diffused ring-like structure can be identified in Fig. 4 ▸(*b*), and most of the intensity is confined in the central bright spot, but the two-lobed structure consistent with the polarization state of the incoming beam is clearly visible. Despite the background diffuse scattering, Fig. 4 ▸(*c*) shows a clear twofold pattern in the differential signal between two images acquired saturating the sample magnetization in the two opposite out-of-plane directions. This pattern cannot be ascribed to the single-shot FEL polarization instability reported above, but it is a clear signature of a rigid rotation of the 180°-period intensity modulation induced by the Faraday effect (Alves *et al.*, 2019 ▸). It is worth noticing that Fig. 4 ▸(*c*) highlights the fact that the Faraday rotation can be measured even very close to the centre of the recorded intensity patterns, where the central bright spot is formed due to the mirror roughness. In order to be more quantitative, Fig. 4 ▸(*d*) shows the polar profile (blue line) extracted from the region of interest marked in Fig. 4 ▸(*b*) by white dashed lines around the intensity minimum labelled by ‘A’. After fitting it with equation (3), a relative Faraday rotation of (4.6 ± 0.4)° can be evaluated with respect to the opposite magnetic saturation state [red line in Fig. 4 ▸(*d*)].

**Figure 5:**
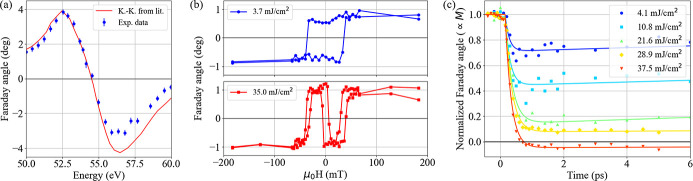
(*a*) Energy scan performed on the FeGd thin film around the Fe *M*_2,3_-edge. The Faraday angle is measured between opposite saturation states, to be directly compared with the hysteresis loop amplitude. The red line shows data taken from von Korff Schmising *et al.* (2020 ▸) and extracted from magnetic circular dichroism measurements via a Kramers-Kronig calculation. (*b*) Hysteresis loops measured at 53.9 eV for two different probing fluences. The experimental points have been obtained by fitting the polar profiles according to equation (3) and by removing from each loop its average value. (*c*) Delay scans measured at 52.8 eV for increasing pump fluences. The curve corresponding to the highest fluence shows magnetization inversion. The solid lines are fits performed according to equation (4).

Having proved the sensitivity of the COMIX polarimeter to small angular variations of the light polarization state, a natural step forward consists of using the device for spectroscopic purposes, *i.e.* varying the beam photon energy to perform a complete scan around the Fe *M*_2,3_-edge. In Fig. 5 ▸(*a*) we report the measured Faraday rotation angle as a function of photon energy (blue dots) compared with similar data extracted from magnetic circular dichroism measurements via a Kramers-Kronig calculation (red continuous line) (von Korff Schmising *et al.*, 2020 ▸). The overall good agreement shown in Fig. 5 ▸(*a*) is a strong indication of the reliability of the COMIX polarimeter as a detection device for XUV Faraday spectroscopy. The small discrepancy at energies above 55 eV can be due to the different Fe and Gd concentrations in the sample measured by von Korff Schmising *et al.* (2020 ▸). The error bars in Fig. 5 ▸(*a*) have been calculated as the square root of the diagonal elements of the covariance matrix returned by the nonlinear fitting procedure of the polar profiles. The average error bar length corresponds to ±0.17°, which, together with the ±0.3° uncertainty estimated from the synchrotron data analysis, sets a limit to the sensitivity of the COMIX device for detecting Faraday rotation.

One important feature of ferrimagnetic TM/RE alloys is the ability to reverse their magnetization after an optical excitation. This process, called ‘all optical switching’, can occur either on ultra-short time scales, where the rotation of the TM sublattice anti-ferromagnetically coupled with the RE species is switched due to a faster demagnetization time constant (Radu *et al.*, 2011 ▸), or on longer time scales (hundreds of ps or ns) where the process is driven by heat diffusion (Stanciu *et al.*, 2006 ▸; Stanciu, Tsukamoto *et al.*, 2007 ▸). A simple way to induce this latter magnetic switching in a multiple exposure measurement is to increase the fluence of the FEL beam at the sample plane, so that the first FEL pulse triggers the rotation process, while the other pulses will probe slower dynamics. Fig. 5 ▸(*b*) shows two hysteresis loops measured at different FEL fluences for a beam photon energy of 53.9 eV. Each one of the experimental points has been obtained by fitting the polar profiles according to equation (3). Two different behaviours can be identified: for low probing fluences, the hysteresis loop has a square-like shape, with a coercive field of 35 mT. When the probing fluence is increased from 3.7 mJ cm^−2^ to 35.0 mJ cm^−2^, the hysteresis loop shows a peculiar structure with the same coercive field, which is in agreement with that reported by Stanciu, Tsukamoto *et al.* (2007 ▸). In that article, changes in the shape of the hysteresis loop were attributed to an increase in the near-infrared pump fluence, and the hysteresis loop was measured at negative probing delays, where the magnetization state is probed long after the excitation. The same is true for the measurement performed by varying the probe fluence without pump, since the effect of the probe on the sample (several ps, typical for a heat-transfer process) is much slower than the interaction between the probe pulse and the sample, on the order of the probe duration (tens of fs). Despite the similarity, in this work we were exciting the sample with a very different photon energy (53.9 eV) with respect to Stanciu, Tsukamoto *et al.* (2007 ▸) (1.55 eV), so some differences in the behaviour and in the fluence thresholds can be expected.

As a last test, the COMIX polarimeter was used for performing near-infrared pump–XUV probe measurements, where the probe was tuned to 52.8 eV. The delay scans obtained at increasing pump fluences are shown in Fig. 5 ▸(*c*). Due to the need for a reference state for measuring polarization rotation variations, each one of the traces combines two delay scans measured while saturating the sample along opposite out-of-plane directions. Only the first 6 ps of the magnetization dynamics are shown, since the main interest is to evaluate to what extent the COMIX polarimeter can capture fine variations of the polarization state. Considering the time range shown in Fig. 5 ▸(*c*), the experimental acquisition time was approximately 1 h per trace. The signature of ultrafast demagnetization is clearly visible in the 0–1 ps range, and the amplitude of the drop increases with increasing pump fluences. At the highest probed fluence, corresponding to 37.5 mJ cm^−2^, the normalized magnetization signal changes sign, which means the ultrafast switching of the magnetization has been achieved in the Fe sub-lattice (Stanciu, Tsukamoto *et al.*, 2007 ▸). In order to extract the Fe demagnetization time τ_*M*_, we fitted the delay scans in Fig. 5 ▸(*c*) with the following function (La-O-Vorakiat *et al.*, 2012 ▸; Caretta *et al.*, 2021 ▸),

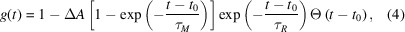

where Δ*A* is the demagnetization amplitude, τ_*R*_ is the magnetization recovery time and Θ(*t* − *t*_0_) is the Heaviside step function centred at *t*_0_. Without including the probe temporal resolution of 70 fs in the fitting function, the obtained values for τ_*M*_ lie in the range 130–220 fs. A detailed discussion on the meaning of these values for FeGd thin films is beyond the scope of this paper, but we notice that the measurements returned values in agreement with literature data (Radu *et al.*, 2011 ▸; von Korff Schmising *et al.*, 2020 ▸).

## 4. Conclusions and outlook

We have presented the characterization of a novel XUV polarimeter based on the simultaneous detection of the whole polarization-dependent reflectivity pattern for XUV radiation. Combining measurements performed at synchrotron radiation and FEL facilities, we have determined a quite wide working energy range in the XUV spectrum, up to 90 eV, *i.e.* covering the energy range containing the *M*_2,3_-edges of Fe, Co and Ni. We have shown that the most important limiting factor that prevents the extension of the working range to higher energies is the surface quality of the mirrors. Using FEL radiation, we have demonstrated a potential application of the COMIX polarimeter for polarization rotation analysis after light–matter interaction with a ferrimagnetic thin film. In particular, we have shown that the COMIX polarimeter can perform spectroscopic studies across the absorption edge of a dichroic magnetic element, and that the device has good sensitivity and stability to detect polarization changes during time-resolved experiments. This prototype version of the COMIX polarimeter can be improved by optimizing the performance of the commercially available aluminium substrate (see Fig. S1 of the supporting information), currently limited to a surface roughness of about 2–3 nm (r.m.s.). For example, we expect that, with a reduction of the roughness to standard optical mirror quality for synchrotron or FEL beamlines (<0.5 nm), the working range can be extended to above 90 eV without changing the Au mirror coating. The required roughness is routinely achieved in high-end XUV optics, even for concave and convex geometries (Trost *et al.*, 2011 ▸). On the other hand, to reach the *N*_4,5_-edges of the more common RE materials (such as Gd, Tb and Dy), XUV multilayer mirrors will be needed for optimizing the transmission of the COMIX polarimeter in an energy range of 140–180 eV. We envisage that, by extending the COMIX working range around the RE absorption edges, the device will become a versatile tool for research in femtomagnetism and for the development of ultrafast spintronics.

## 5. Related literature

The following references, not cited in the main body of the paper, have been cited in the supporting information: Willems *et al.* (2015 ▸); Yao *et al.* (2020 ▸).

## Supplementary Material

Sections S1 to S3; Figures S1 to S3. DOI: 10.1107/S1600577522004027/ve5156sup1.pdf

Intensity patterns. DOI: 10.1107/S1600577522004027/ve5156sup2.avi

## References

[bb1] Allaria, E., Appio, R., Badano, L., Barletta, W. A., Bassanese, S., Biedron, S. G., Borga, A., Busetto, E., Castronovo, D., Cinquegrana, P., Cleva, S., Cocco, D., Cornacchia, M., Craievich, P., Cudin, I., D’Auria, G., Dal Forno, M., Danailov, M. B., De Monte, R., De Ninno, G., Delgiusto, P., Demidovich, A., Di Mitri, S., Diviacco, B., Fabris, A., Fabris, R., Fawley, W., Ferianis, M., Ferrari, E., Ferry, S., Froehlich, L., Furlan, P., Gaio, G., Gelmetti, F., Giannessi, L., Giannini, M., Gobessi, R., Ivanov, R., Karantzoulis, E., Lonza, M., Lutman, A., Mahieu, B., Milloch, M., Milton, S. V., Musardo, M., Nikolov, I., Noe, S., Parmigiani, F., Penco, G., Petronio, M., Pivetta, L., Predonzani, M., Rossi, F., Rumiz, L., Salom, A., Scafuri, C., Serpico, C., Sigalotti, P., Spampinati, S., Spezzani, C., Svandrlik, M., Svetina, C., Tazzari, S., Trovo, M., Umer, R., Vascotto, A., Veronese, M., Visintini, R., Zaccaria, M., Zangrando, D. & Zangrando, M. (2012). *Nat. Photon.* **6**, 699–704.

[bb2] Allaria, E., Diviacco, B., Callegari, C., Finetti, P., Mahieu, B., Viefhaus, J., Zangrando, M., De Ninno, G., Lambert, G., Ferrari, E., Buck, J., Ilchen, M., Vodungbo, B., Mahne, N., Svetina, C., Spezzani, C., Di Mitri, S., Penco, G., Trovó, M., Fawley, W. M., Rebernik, P. R., Gauthier, D., Grazioli, C., Coreno, M., Ressel, B., Kivimäki, A., Mazza, T., Glaser, L., Scholz, F., Seltmann, J., Gessler, P., Grünert, J., De Fanis, A., Meyer, M., Knie, A., Moeller, S. P., Raimondi, L., Capotondi, F., Pedersoli, E., Plekan, O., Danailov, M. B., Demidovich, A., Nikolov, I., Abrami, A., Gautier, J., Lüning, J., Zeitoun, P. & Giannessi, L. (2014). *Phys. Rev. X*, **4**, 041040.

[bb3] Alves, C., Lambert, G., Malka, V., Hehn, M., Malinowski, G., Hennes, M., Chardonnet, V., Jal, E., Lüning, J. & Vodungbo, B. (2019). *Phys. Rev. B*, **100**, 144421.

[bb4] Ayuso, D., Neufeld, O., Ordonez, A. F., Decleva, P., Lerner, G., Cohen, O., Ivanov, M. & Smirnova, O. (2019). *Nat. Photonics*, **13**, 866–871.

[bb5] Bergeard, N., López-Flores, V., Halté, V., Hehn, M., Stamm, C., Pontius, N., Beaurepaire, E. & Boeglin, C. (2014). *Nat. Commun.* **5**, 3466. 10.1038/ncomms4466 24614016

[bb6] Capotondi, F., Pedersoli, E., Mahne, N., Menk, R. H., Passos, G., Raimondi, L., Svetina, C., Sandrin, G., Zangrando, M., Kiskinova, M., Bajt, S., Barthelmess, M., Fleckenstein, H., Chapman, H. N., Schulz, J., Bach, J., Frömter, R., Schleitzer, S., Müller, L., Gutt, C. & Grübel, G. (2013). *Rev. Sci. Instrum.* **84**, 051301. 10.1063/1.4807157 23742525

[bb7] Caretta, A., Laterza, S., Bonanni, V., Sergo, R., Dri, C., Cautero, G., Galassi, F., Zamolo, M., Simoncig, A., Zangrando, M., Gessini, A., Zilio, S. D., Flammini, R., Moras, P., Demidovich, A., Danailov, M., Parmigiani, F. & Malvestuto, M. (2021). *Struct. Dyn.* **8**, 034304. 10.1063/4.0000104 PMC8214468 34169118

[bb8] Danailov, M. B., Bencivenga, F., Capotondi, F., Casolari, F., Cinquegrana, P., Demidovich, A., Giangrisostomi, E., Kiskinova, M. P., Kurdi, G., Manfredda, M., Masciovecchio, C., Mincigrucci, R., Nikolov, I. P., Pedersoli, E., Principi, E. & Sigalotti, P. (2014). *Opt. Express*, **22**, 12869. 10.1364/OE.22.012869 24921484

[bb9] Derossi, A., Lama, F., Piacentini, M., Prosperi, T. & Zema, N. (1995). *Rev. Sci. Instrum.* **66**, 1718–1720.

[bb10] Desjardins, K., Medjoubi, K., Sacchi, M., Popescu, H., Gaudemer, R., Belkhou, R., Stanescu, S., Swaraj, S., Besson, A., Vijayakumar, J., Pautard, S., Noureddine, A., Mercère, P., Da Silva, P., Orsini, F., Menneglier, C. & Jaouen, N. (2020). *J. Synchrotron Rad.* **27**, 1577–1589. 10.1107/S160057752001262X 33147182

[bb11] Ferrari, E., Allaria, E., Buck, J., De Ninno, G., Diviacco, B., Gauthier, D., Giannessi, L., Glaser, L., Huang, Z., Ilchen, M., Lambert, G., Lutman, A. A., Mahieu, B., Penco, G., Spezzani, C. & Viefhaus, J. (2015). *Sci. Rep.* **5**, 13531. 10.1038/srep13531 PMC4551986 26314764

[bb12] Fleischer, A., Kfir, O., Diskin, T., Sidorenko, P. & Cohen, O. (2014). *Nat. Photon.* **8**, 543–549.

[bb13] Fowles, G. R. (1989). *Introduction to Modern Optics.* New York: Dover Publications.

[bb14] Frassetto, F., Zuppella, P., Samparisi, F., Fabris, N. & Poletto, L. (2019). *Proc. SPIE*, **11038**, 110380M.

[bb15] Henke, B. L., Gullikson, E. M. & Davis, J. C. (1993). *At. Data Nucl. Data Tables*, **54**, 181–342.

[bb16] Hofherr, M., Häuser, S., Dewhurst, J. K., Tengdin, P., Sakshath, S., Nembach, H. T., Weber, S. T., Shaw, J. M., Silva, T. J., Kapteyn, H. C., Cinchetti, M., Rethfeld, B., Murnane, M. M., Steil, D., Stadtmüller, B., Sharma, S., Aeschlimann, M. & Mathias, S. (2020). *Sci. Adv.* **6**, eaay8717. 10.1126/sciadv.aay8717 PMC6968944 32010774

[bb17] Kfir, O., Grychtol, P., Turgut, E., Knut, R., Zusin, D., Popmintchev, D., Popmintchev, T., Nembach, H., Shaw, J. M., Fleischer, A., Kapteyn, H., Murnane, M. & Cohen, O. (2015). *Nat. Photon.* **9**, 99–105.

[bb18] Khorsand, A. R., Savoini, M., Kirilyuk, A., Kimel, A. V., Tsukamoto, A., Itoh, A. & Rasing, Th. (2013). *Phys. Rev. Lett.* **110**, 107205. 10.1103/PhysRevLett.110.107205 23521292

[bb19] Kirilyuk, A., Kimel, A. V. & Rasing, T. (2010). *Rev. Mod. Phys.* **82**, 2731–2784.

[bb20] Korff Schmising, C. von, Weder, D., Noll, T., Pfau, B., Hennecke, M., Strüber, C., Radu, I., Schneider, M., Staeck, S., Günther, C. M., Lüning, J., Merhe, A. E. D., Buck, J., Hartmann, G., Viefhaus, J., Treusch, R. & Eisebitt, S. (2017). *Rev. Sci. Instrum.* **88**, 053903. 10.1063/1.4983056 28571434

[bb21] Korff Schmising, C. von, Willems, F., Sharma, S., Yao, K., Borchert, M., Hennecke, M., Schick, D., Radu, I., Strüber, C., Engel, D. W., Shokeen, V., Buck, J., Bagschik, K., Viefhaus, J., Hartmann, G., Manschwetus, B., Grunewald, S., Düsterer, S., Jal, E., Vodungbo, B., Lüning, J. & Eisebitt, S. (2020). *Appl. Sci.* **10**, 7580.

[bb22] Lan, G. & Twa, M. D. (2019). *Opt. Express*, **27**, 5048. 10.1364/OE.27.005048 PMC6410919 30876110

[bb23] La-O-Vorakiat, C., Turgut, E., Teale, C. A., Kapteyn, H. C., Murnane, M. M., Mathias, S., Aeschlimann, M., Schneider, C. M., Shaw, J. M., Nembach, H. T. & Silva, T. J. (2012). *Phys. Rev. X*, **2**, 011005.

[bb24] Lutman, A. A., MacArthur, J. P., Ilchen, M., Lindahl, A. O., Buck, J., Coffee, R. N., Dakovski, G. L., Dammann, L., Ding, Y., Dürr, H. A., Glaser, L., Grünert, J., Hartmann, G., Hartmann, N., Higley, D., Hirsch, K., Levashov, Y. I., Marinelli, A., Maxwell, T., Mitra, A., Moeller, S., Osipov, T., Peters, F., Planas, M., Shevchuk, I., Schlotter, W. F., Scholz, F., Seltmann, J., Viefhaus, J., Walter, P., Wolf, Z. R., Huang, Z. & Nuhn, H.-D. (2016). *Nat. Photon.* **10**, 468–472.

[bb25] Mertins, H., Oppeneer, P. M., Valencia, S., Gudat, W., Senf, F. & Bressler, P. R. (2004). *Phys. Rev. B*, **70**, 235106.

[bb26] Palmer, B. A., Morte-Ródenas, A., Kariuki, B. M., Harris, K. D. M. & Collins, S. P. (2011). *J. Phys. Chem. Lett.* **2**, 2346–2351.

[bb27] Rabinovitch, K., Canfield, L. R. & Madden, R. P. (1965). *Appl. Opt.* **4**, 1005.

[bb28] Radu, I., Vahaplar, K., Stamm, C., Kachel, T., Pontius, N., Dürr, H. A., Ostler, T. A., Barker, J., Evans, R. F. L., Chantrell, R. W., Tsukamoto, A., Itoh, A., Kirilyuk, A., Rasing, Th. & Kimel, A. V. (2011). *Nature*, **472**, 205–208. 10.1038/nature09901 21451521

[bb29] Raimondi, L., Manfredda, M., Mahne, N., Cocco, D., Capotondi, F., Pedersoli, E., Kiskinova, M. & Zangrando, M. (2019). *J. Synchrotron Rad.* **26**, 1462–1472. 10.1107/S1600577519007938 31490133

[bb30] Raimondi, L., Svetina, C., Mahne, N., Cocco, D., Abrami, A., De Marco, M., Fava, C., Gerusina, S., Gobessi, R., Capotondi, F., Pedersoli, E., Kiskinova, M., De Ninno, G., Zeitoun, P., Dovillaire, G., Lambert, G., Boutu, W., Merdji, H., Gonzalez, A. I., Gauthier, D. & Zangrando, M. (2013). *Nucl. Instrum. Methods Phys. Res. A*, **710**, 131–138.

[bb31] Sacchi, M., Spezzani, C., Torelli, P., Avila, A., Delaunay, R. & Hague, C. F. (2003). *Rev. Sci. Instrum.* **74**, 2791–2795.

[bb32] Schäfers, F., Mertins, H.-C., Gaupp, A., Gudat, W., Mertin, M., Packe, I., Schmolla, F., Di Fonzo, S., Soullié, G., Jark, W., Walker, R., Le Cann, X., Nyholm, R. & Eriksson, M. (1999). *Appl. Opt.* **38**, 4074–4088. 10.1364/ao.38.004074 18323885

[bb33] Stanciu, C. D., Hansteen, F., Kimel, A. V., Kirilyuk, A., Tsukamoto, A., Itoh, A. & Rasing, Th. (2007). *Phys. Rev. Lett.* **99**, 047601. 10.1103/PhysRevLett.99.047601 17678404

[bb34] Stanciu, C. D., Kimel, A. V., Hansteen, F., Tsukamoto, A., Itoh, A., Kirilyuk, A. & Rasing, Th. (2006). *Phys. Rev. B*, **73**, 220402.

[bb35] Stanciu, C. D., Tsukamoto, A., Kimel, A. V., Hansteen, F., Kirilyuk, A., Itoh, A. & Rasing, Th. (2007). *Phys. Rev. Lett.* **99**, 217204. 10.1103/PhysRevLett.99.217204 18233247

[bb36] Trost, M., Schröder, S., Feigl, T., Duparré, A. & Tünnermann, A. (2011). *Appl. Opt.* **50**, C148–C153. 10.1364/AO.50.00C148 21460930

[bb37] Tschentscher, T., Bressler, C., Grünert, J., Madsen, A., Mancuso, A., Meyer, M., Scherz, A., Sinn, H. & Zastrau, U. (2017). *Appl. Sci.* **7**, 592.

[bb38] Valencia, S., Gaupp, A., Gudat, W., Mertins, H.-C., Oppeneer, P. M., Abramsohn, D. & Schneider, C. M. (2006). *New J. Phys.* **8**, 254.

[bb39] Viefhaus, J., Scholz, F., Deinert, S., Glaser, L., Ilchen, M., Seltmann, J., Walter, P. & Siewert, F. (2013). *Nucl. Instrum. Methods Phys. Res. A*, **710**, 151–154.

[bb887] Willems, F., Smeenk, C. T. L., Zhavoronkov, N., Kornilov, O., Radu, I., Schmidbauer, M., Hanke, M., von Korff Schmising, C., Vrakking, M. J. J. & Eisebitt, S. (2015). *Phys. Rev. B*, **92**, 220405.

[bb40] Willems, F., von Korff Schmising, C., Strüber, C., Schick, D., Engel, D. W., Dewhurst, J. K., Elliott, P., Sharma, S. & Eisebitt, S. (2020). *Nat. Commun.* **11**, 871. 10.1038/s41467-020-14691-5 PMC7018696 32054855

[bb41] Yamamoto, S. & Matsuda, I. (2017). *Appl. Sci.* **7**, 662.

[bb42] Yamamoto, Sh., Taguchi, M., Someya, T., Kubota, Y., Ito, S., Wadati, H., Fujisawa, M., Capotondi, F., Pedersoli, E., Manfredda, M., Raimondi, L., Kiskinova, M., Fujii, J., Moras, P., Tsuyama, T., Nakamura, T., Kato, T., Higashide, T., Iwata, S., Yamamoto, S., Shin, S. & Matsuda, I. (2015). *Rev. Sci. Instrum.* **86**, 083901. 10.1063/1.4927828 26329205

[bb886] Yao, K., Willems, F., von Korff Schmising, C., Strüber, C., Hessing, P., Pfau, B., Schick, D., Engel, D., Gerlinger, K., Schneider, M. & Eisebitt, S. (2020). *Rev. Sci. Instrum.* **91**, 093001. 10.1063/5.0013928 33003828

