# Supplementary material for: The COMIX polarimeter: a compact device for XUV polarization analysis
Source: J Synchrotron Radiat. 2022 May 19;29(Pt 4):969–77. doi: 10.1107/S1600577522004027 (PMC9255573; doi:10.1107/S1600577522004027)
Supplement: Supplementary file 1 [file s-29-00969-sup1.pdf]

JOURNAL OF SYNCHROTRON RADIATION

Volume 29 (2022)

Supporting information for article:

The COMIX polarimeter: a compact device for XUV polarization analysis

Matteo Pancaldi, Christian Strüber, Bertram Friedrich, Emanuele Pedersoli, Dario De Angelis, Ivaylo P. Nikolov, Michele Manfredda, Laura Foglia, Sergiy Yulin, Carlo Spezzani, Maurizio Sacchi, Stefan Eisebitt, Clemens von Korff Schmising and Flavio Capotondi

S1. Conical Substrate Roughness

As described and reported in Fig. 2 of the main text of the article, diffuse scattering due to mirror roughness is the limiting factor affecting the COMIX performance, especially at high photon energy (> 90 eV). According to the energy dependence of the transmitted photons, we inferred from Fig. 2(h) a mirror roughness of about 2 – 3 nm. For further development of the device, the main source affecting the mirror roughness of the prototype should be understood. In this context, we performed atomic force microscopy (AFM) images of the pristine aluminium conical substrate, provided by Thorlabs Inc., before gold coating deposition. Fig. S1(a) shows an example of AFM morphology of the conical substrate on an area of 5 μm × 10 μm. From the analysis of AFM height profiles, Fig. S1(b), we estimate a roughness of about 2 nm (rms) for the aluminium substrate. According to such results, we expect that improving the surface roughness of the conical substrates, e.g. using glass substrates (like fused silica, silica, Zerodur) instead of a metallic one, will have beneficial effects on the performance of the COMIX polarimeter.

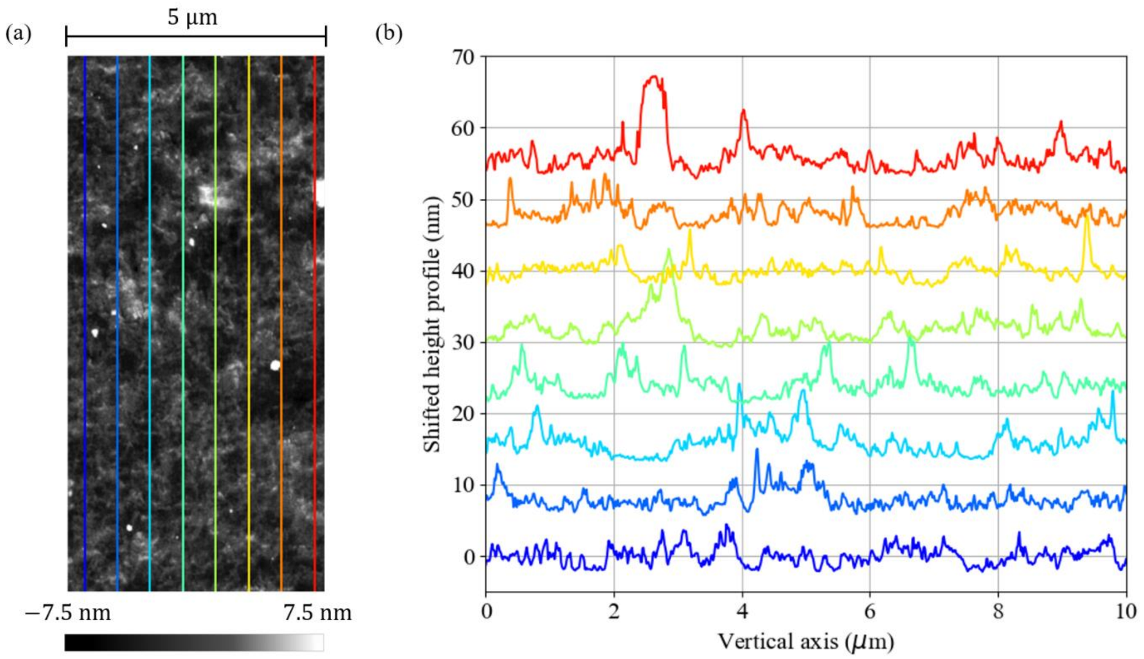

Figure S1 (a) AFM image of the aluminium conical substrate for the gold-coated mirror, which shows a high-spatial frequency roughness of about 2 nm (rms). The inspected area was 5 μm × 10 μm. (b) Selected line profiles on different positions of the image in panel (a).

S2. Polarisation Control

In this section, we add further information on the ability of the COMIX polarimeter to analyse the polarisation state of the incoming radiation. As discussed in Section 2 of the main article, the evaluation of polarisation state and the degree of circular polarisation was partially limited by the alignment of the COMIX apex tip and by the spurious off-axis radiation generated by the beamline electromagnetic elliptical wiggler. Here we report similar measurements performed on a laser-driven high harmonic generation (HHG) source operating in the XUV spectral range, between approximately 40 and 72 eV (Yao *et al.*, 2020). In this experiment, the degree of polarisation was controlled by phase retardation of in-plane and out-of-plane polarisation components upon reflection off four metallic mirrors (Willems *et al.*, 2015; von Korff Schmising *et al.*, 2017), and another COMIX polarimeter prototype was used. Differently from the experiments reported in the main text, due to geometrical constraints, the working distance between the COMIX polarimeter and the CCD detector was reduced to 50 mm, and the radiation generated from the HHG source was energetically monochromatized by selecting a single harmonic emission peak at 54 eV with a multilayer mirror.

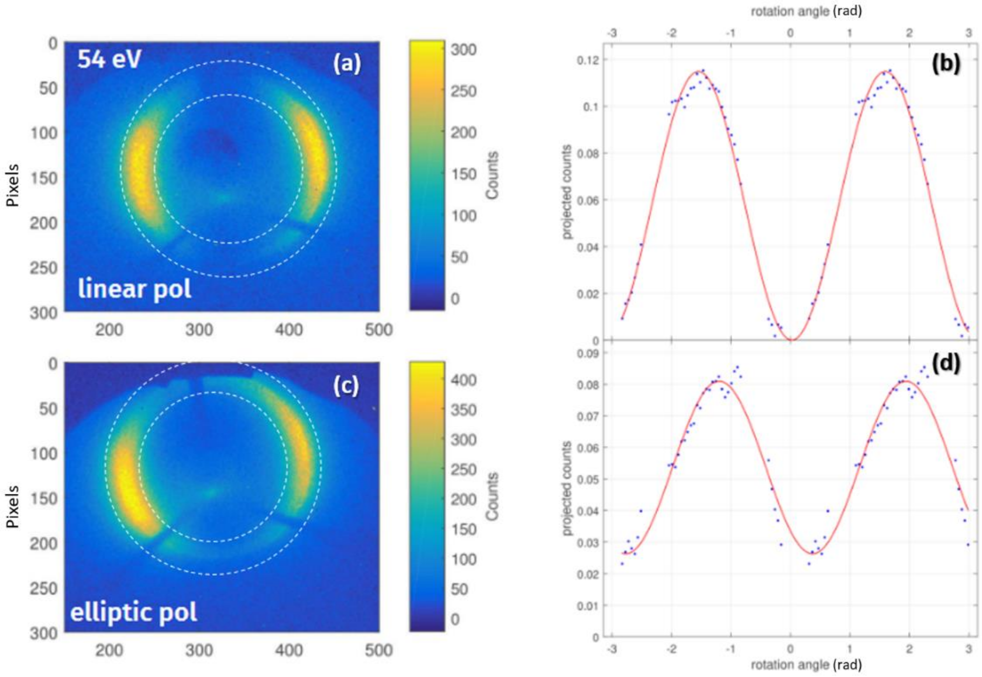

Figure S2 (a)-(c) COMIX intensity patterns recorded at a fixed photon energy of 54 eV for linear and elliptical polarisations. (b)-(d) Polar profiles and relative polarisation fits.

Figures S2(a) and S2(b) show the intensity distribution for linear vertical polarisation and its polar profile extracted from the region contained between the dashed circles. The red line in Fig. S2(b) represents the best fit to the experimental data using the polar function $f(\vartheta)$ reported in Eq. (3). On the other hand, Fig. S2(c) shows the intensity distribution in case of an elliptical polarisation, which is induced by applying a phase shift using the four-mirror polariser. Comparing the fits, the polar profile reported in Fig. S2(d) shows a counter-clockwise rotation of the light polarisation axis of 20 deg, and

the generation of an elliptical polarisation with an ellipticity of 0.66 (degree of circular polarisation of approx. 49%). Discrepancies to the previously reported degree of circular polarisation of 75% which have been identified by this measurement were traced back to contamination of the phase retardation mirrors that have been subsequently replaced.

S3. Statistical Analysis of Single-shot FEL Polarisation

Before performing the magneto-optical experiments described in Section 3 of the article, we used the COMIX polarimeter for performing single-shot polarisation stability analysis of the linear horizontal FEL radiation emitted by the FERMI source. Figures S3(a)-(c) show a set of single-shot images of the COMIX intensity distribution at a photon energy of 53.6 eV. The intensity distribution shows the typical two-lobe pattern for linear horizontal polarisation. Figures S3(d) and S3(e) show the polar profile extracted close to the intensity minimum labelled by “A” and its position fluctuations for 20 successive FEL pulses, respectively. The fits of the minimum position of the experimental data with the model function $f(\vartheta)$ reported in Eq. (3) show a polarisation stability of the electric field vector along the linear horizontal polarisation direction of approximately 0.8 deg in FWHM (light-blue dashed lines in Fig. S3(e)).

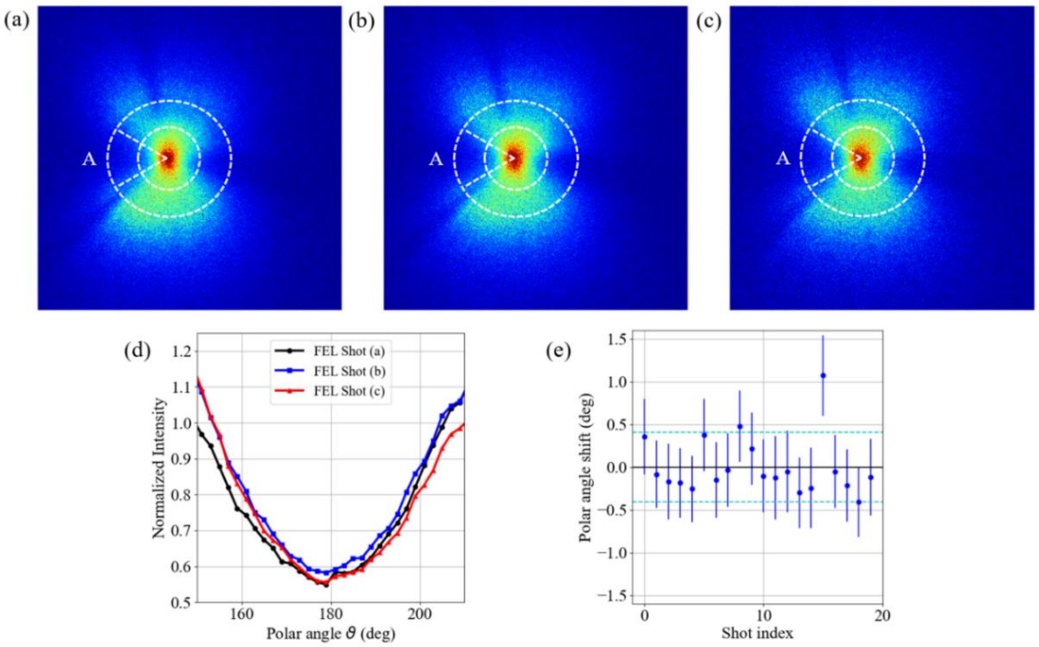

Figure S3 (a)-(c) Intensity patterns recorded without sample from three different single FEL shots at 53.6 eV (linear horizontal polarisation). The three images share the same colour bar and have been normalized to the corresponding shot intensity. (d) Polar profiles obtained in a ±30 deg range around the intensity minimum labelled by “A” for images (a)-(c). The nearly identical position of the plots’ minima demonstrates the polarisation stability of the source. (e) Relative fluctuation of the position of the intensity minimum at $\vartheta \approx 180$ deg for 20 successive single FEL shots.

References

von Korff Schmising, C., Weder, D., Noll, T., Pfau, B., Hennecke, M., Strüber, C., Radu, I., Schneider, M., Staeck, S., Günther, C. M., Lüning, J., Merhe, A. el dine, Buck, J., Hartmann, G., Viefhaus, J., Treusch, R. & Eisebitt, S. (2017). *Review of Scientific Instruments*. **88**, 053903.

Willems, F., Smeenk, C. T. L., Zhavoronkov, N., Kornilov, O., Radu, I., Schmidbauer, M., Hanke, M., von Korff Schmising, C., Vrakking, M. J. J. & Eisebitt, S. (2015). *Phys. Rev. B*. **92**, 220405.

Yao, K., Willems, F., von Korff Schmising, C., Strüber, C., Hessing, P., Pfau, B., Schick, D., Engel, D., Gerlinger, K., Schneider, M. & Eisebitt, S. (2020). *Review of Scientific Instruments*. **91**, 093001.
